# Long-Term Outcomes and Factors Associated with Mortality in Patients with Moderate to Severe Pulmonary Hypertension in Kenya

**DOI:** 10.5334/gh.384

**Published:** 2020-02-06

**Authors:** Mzee Ngunga, Abdulaziz Mansur Abeid, Jeilan Mohamed, Anders Barasa

**Affiliations:** 1 Aga Khan University Hospital, Nairobi, KE

**Keywords:** pulmonary hypertension, long-term outcomes, prognostic factors

## Abstract

**Background:**

Pulmonary hypertension is poorly studied in Africa. The long-term survival rates and prognostic factors associated with mortality in patients with moderate to severe pulmonary hypertension (PH) in Africa are not well described.

**Objectives:**

To determine the causes of moderate to severe PH in patients seen in contemporary hospital settings, determine the patients’ one-year survival and the factors associated with mortality following standard care.

**Methods:**

A retrospective review of patients diagnosed with moderate to severe PH at Aga Khan University Hospital (AKUHN) from August 2014 to July 2017 was carried out. Clinical and outcome data were collected from medical records and the hospital mortality database. Telephone interviews were conducted for patients who died outside the hospital. Survival analysis was done using Kaplan-Meier, and log-rank tests were used to assess differences between subgroups. Cox regression modelling with multivariable adjustment was used to identify factors associated with all-cause mortality.

**Results:**

A total of 659 patients with moderate to severe PH were enrolled. Median follow-up time was 626 days. The survival rates of the patients at 1 and 2 years were 73.8% and 65.9%, respectively. The following variables were significantly associated with mortality: diabetes mellitus [adjusted HR 1.52, 95% CI (1.14–2.01)], WHO functional class III/IV [adjusted HR 3.49, 95% CI (2.46–4.95)], atrial fibrillation [adjusted HR 1.53, 95% CI (1.08–2.17)], severe PH [adjusted HR 1.72, 95% CI (1.30–2.27)], right ventricular dysfunction [adjusted HR 2.42, 95% CI (1.76–3.32)] and left ventricular dysfunction [adjusted HR 1.91, 95% CI (1.36–2.69)]. Obesity [adjusted HR 0.68, 95% CI (0.50–0.93)] was associated with improved survival.

**Conclusion:**

Pulmonary hypertension is associated with poor long-term outcomes in African patients. Identification of prognostic factors associated with high-risk patients will assist in patient management and potentially improved outcomes.

## Introduction

There is an increasing burden of pulmonary hypertension around the world. A recent global review postulates that approximately 1% of the global population is affected [1]. In addition, approximately one-tenth of those aged 65 years of age and above are thought to suffer pulmonary hypertension [1]. It is, however, important to note that PH increasingly affects people of all ages both in high-income and low to middle-income countries. Indeed, 80% of the burden of PH is felt in low and middle-income countries [1]. In Kenya, the prevalence of PH has only been studied in selected patient populations with specific diseases and it ranges between 5.5% and 49.4% [2,3].

Pulmonary hypertension has various aetiologies [4]. This results in variation in the clinical profile of PH between patients and across different regions of the world [5,6]. The prognosis of PH patients is dependent of different factors, and similar to the clinical profiles, they vary greatly [7]. Previous studies have demonstrated that diseases and risk factors associated with PH in low and middle-income countries are highly prevalent in Kenya. These include: HIV, rheumatic heart disease, sickle cell disease and schistosomiasis [8,9]. The pervasion of poor lifestyle choices and habits in Kenya, as well as in many other low and middle-income countries, means that the prevalence of hypertensive heart disease and chronic obstructive pulmonary disease is on the rise [10]. It then follows that there is more likely than not an increased risk of PH in Kenya that needs to be fully investigated.

Studies show that PH-related mortality rates are high, with associated low survival rates, especially for those with moderate to severe PH. A study of the the Registry to Evaluate Early and Long-term PAH Disease Management (REVEAL) in the United States found a 5 year-survival rate of 27% among patients with PH [11]. A study done in four countries in sub-Saharan Africa reported a 6-month mortality rate of 21% [5]. However, the long-term outcomes of African patients with PH have not been studied. In addition, the high PH mortality points to a need to understand and characterize prognostic factors associated with mortality or survival to better inform prevention and management efforts. This study, therefore, sought to determine the 1-year and 3-year survival rates and prognostic factors associated with all-cause mortality among patients with moderate to severe PH attending a tertiary referral hospital in Kenya.

## Methods

### Study Design

This was a retrospective hospital-based cohort study of patients diagnosed with moderate to severe PH between August 2014 and July 2017. The study was carried out at Aga Khan University Hospital, Nairobi (AKUHN), a private non-profit tertiary referral hospital serving patients from Eastern Africa.

The study population was drawn from the AKUHN echo database of patients with a first echocardiographic diagnosis of moderate to severe PH from August 2014 to July 2017. Case definition was based on pulmonary artery systolic pressure (PASP) as follows: 45–59 mm Hg (moderate) and ≥60 mm Hg (severe) [12,13]. Cases with missing clinical variables of interest were excluded. Other exclusion criteria included pulmonary valve peak velocity above 3 m/s and acute elevation of pulmonary pressure due to acute pulmonary embolism or acute lung pathologies.

### Data collection

Data was collected from patient medical records using EPI INFO software version 7.2.1.0. Clinical variables were obtained from the medical records. Etiology of PH was defined as the one assigned by the primary physician after review of the clinical data and investigations available. Unclear causes from the available data would deem the cause of PH as ‘unclassified’. A 12-lead electrocardiogram was analyzed for the presence of atrial fibrillation based on American Heart Association (AHA) and Heart Rhythm Society guidelines [14].

Echocardiographic studies were performed using the GE Vivid 7 Dimension and Vivid Q Ultrasound machines utilizing a 3–5 MHz sector array transducer probe. Standard parasternal, apical and subcostal views were obtained. 2D-echocardiography, M-mode, and Doppler studies were performed on all patients. All echocardiograms were reviewed and validated by a certified consultant cardiologist.

Echocardiographic variables were defined according to the American Society of Echocardiography (ASE) guidelines [15,16]. A simplified Bernoulli equation was used to estimate the PASP using the tricuspid regurgitant velocity (TRV) and right atrial pressures. Right atrial pressures were estimated by the use of size and extent of collapsibility of the inferior vena cava (IVC). Tricuspid annular plane systolic excursion (TAPSE) was used to assess RV function; it was measured by M-mode echocardiography with the cursor favorably positioned with the direction of the tricuspid lateral annulus in the apical four-chamber view. RV dysfunction was defined as TAPSE < 17 mm. The left ventricular ejection fraction was estimated using the Simpson method. Color flow Doppler allowed for the detection and grading severity of regurgitant lesions. Doppler measurements to determine gradients across valves were used to assess the severity of stenotic lesions. Significant valvular heart disease was defined as any moderate to severe stenosis or regurgitation. The presence of rheumatic valvular changes was captured by assessing valve morphology on 2D echocardiography.

Telephone interviews were conducted when survival status was unknown from medical records. Patients were called directly at least two times, and if unsuccessful, the next of kin was contacted to provide outcome data. Where unavailable, censoring was done at the last date of hospital contact.

### Data Analysis

Data analysis was carried out using SPSS version 24. Frequencies and proportions were used to summarise categorical data, with bivariate relationships analysed using Pearson’s Chi-square. For continuous data means and standard deviations or medians and interquartile ranges were provided depending on the normality of the data. Comparison of means and medians was done using t-tests and Mann-Whitney U tests, respectively.

Kaplan-Meier estimates were used in analysis of survival rates. The date of the first echocardiogram with PASP ≥ 45 mmHg was taken as the start date. Patients still alive at the end of the follow-up period or lost to follow-up were censored. Duration of follow up was calculated from the time of first echocardiographic diagnosis of PH to death or date of censoring. Reverse Kaplan-Meier method was utilized in calculation of the median follow-up time. Kaplan-Meier analysis method was used in the analysis of the survival rates and survival curve differences through the log-rank test.

Cox proportional hazard regression was modelled to capture the factors that were correlated with mortality among patients with moderate to severe PH. First, unadjusted Hazard Ratios were analysed by the use of univariate cox regression. Variables significant at p < 0.05 at this stage were analysed together using multivariate Cox regression to estimate adjusted Hazard Ratios with 95% confidence intervals. A two-tailed p < 0.05 was considered significant.

### Ethical Considerations

The study received approval from the Aga Khan University Ethics Review Committee. Respondents gave verbal consent over the telephone after the study and its aims were explained to them. Study data was anonymised and stored as per institutional policies to ensure confidentiality. The study protocol conforms to the ethical guidelines of the 1975 Declaration of Helsinki as reflected in a prior approval by the institution’s human research committee.

## Results

### Study Cohort

**Figure 1:**
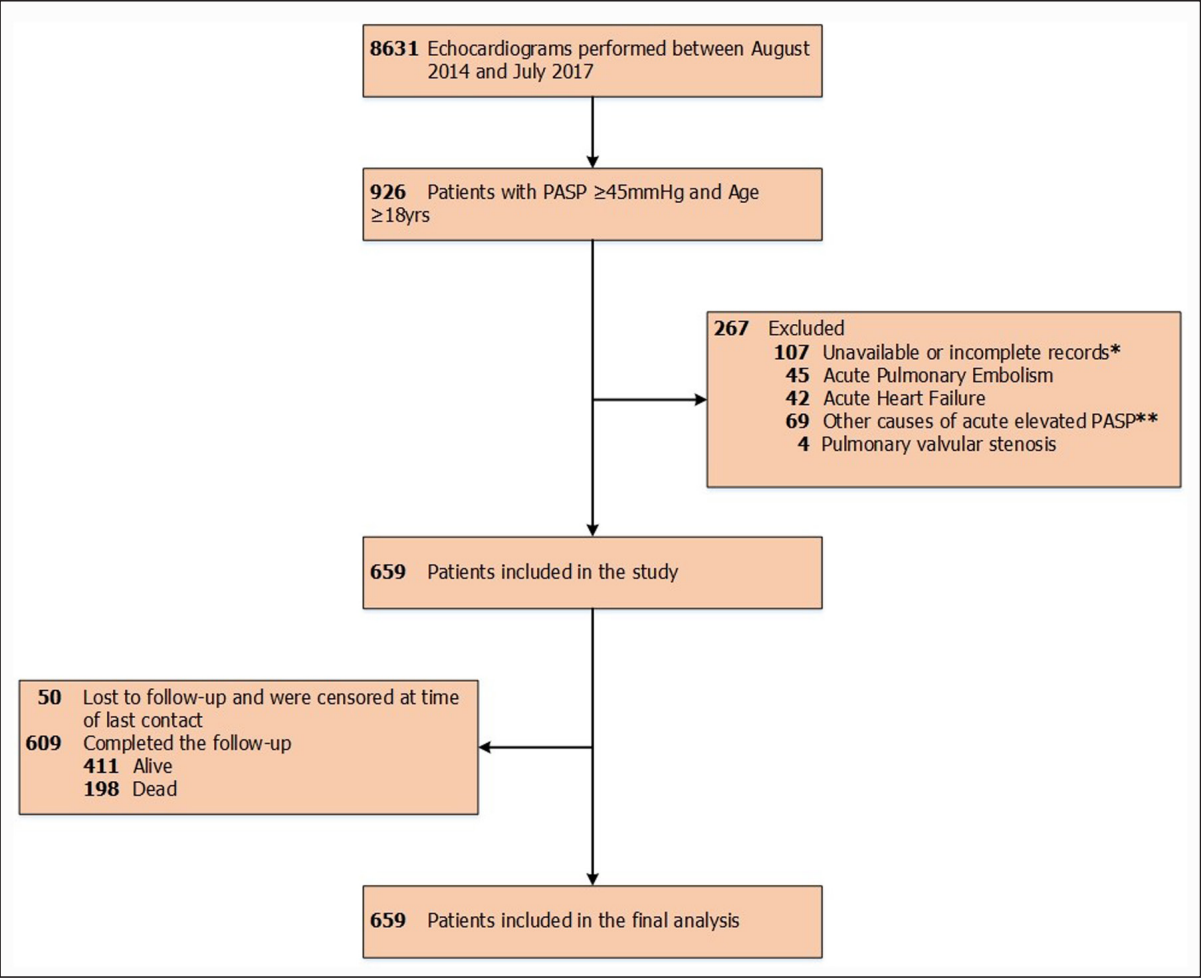
Flow diagram showing the selection of study cohort. * Cases with absent clinical data or electrocardiogram. ** Patients with transient elevated PASP due to acute respiratory infections, acute respiratory distress syndrome and positive pressure mechanical ventilation.

A total of 8631 echocardiograms were carried out from August 2014 to July 2017 and 926 patients were found to have PASP ≥ 45 mmHg, of whom 267 patients did not meet the inclusion criteria, resulting in a cohort of 659 patients who were included in the final analysis as shown in Figure 1.

### Demographic and Clinical characteristics

The average age of respondents was 66 years (SD = 17.45), and majority were female (55.1%) and of black African race (80%). The most common comorbidity was systemic arterial hypertension (69.7%) followed by heart failure (52.2%). HIV was tested in 206 patients, of whom 28 (13.6%) were positive. Fifty-seven percent of the cohort were in functional class III or IV. Table 1 provides the summary of baseline patient characteristics.

**Table 1:** Baseline Demographic and Clinical Characteristics.

	All patients N = 659

**Demographics**	
Age (years)	65.72 ± 17.45
Gender, n (%)	
Females	363 (55.1)
Males	296 (44.9)
Race	
Blacks, n (%)	527 (80.0)
Non-Blacks, n (%)	132 (20.0)
**Comorbidities**	
Systemic Arterial Hypertension, n (%)	459 (69.7)
Diabetes Mellitus, n (%)	224 (34.0)
Smoking, n (%)	66 (10.0)
Heart failure, n (%)	344 (52.2)
COPD, n (%)	78 (11.8)
HIV, n (%) N = 206	28 (13.6)
Systolic BP (mmHg), median (IQR)	82 (72–94)
Systolic BP (mmHg), median (IQR)	129 (111–147)
Weight (kg)	76.23 ± 18.82
Body Mass Index, median (IQR)	28 (24–32)
Underweight, BMI < 18.5	22 (3.3%)
Normal, BMI 18.5–24.9	161 (24.4%)
Overweight, BMI 25.0–29.9	235 (35.7%)
Stage I obesity, BMI 30.0–34.9	136 (20.6%)
Stage II obesity, BMI 35.0–39.9	59 (9%)
Extreme Obesity, BMI ≥ 40.0	43 (6.5%)
**WHO functional class, n (%)**	
Class I/II	192 (43.5)
Class III/V	249 (56.5)

* Plus-minus values are means ± SD. IQR, interquartile range; COPD, chronic obstructive pulmonary disease; HIV, human immunodeficiency virus; BP, blood pressure; WHO, world health organization.

### Echocardiographic and Electrocardiographic Findings

On electrocardiogram, 99 (15%) patients had atrial fibrillation. The median PASP was 56 mmHg (25th–75th percentiles: 49–68 mmHg) and TAPSE was 18 mm (25th–75th percentiles: 15–20 mm). About 28.2% of patients had LV ejection fraction below 40%, while 28.7% had significant valvular abnormalities. Surprisingly, rheumatic and congenital heart diseases were present in similar proportions (5.6% vs. 5.8%). The summary of the other findings is shown in Table 2.

**Table 2:** Electrocardiographic and Echocardiographic findings.

	All patients (N = 650)

**Electrocardiographic findings**	
Atrial Fibrillation, n (%)	99 (15.0)
**Echocardiographic findings**	
TRV (ms), median (IQR)	3.42 (3.22–3.79)
RAP (mmHg)	9.60 ± 5.28
PASP (mmHg), median (IQR)	56 (49–68)
TAPSE (mm) (N = 580), median (IQR)	18 (15–20)
LVEF (%), median (IQR)	55 (38–60)
LVEF < 40%	186 (28.2)
Congenital Heart Disease, n (%)	38 (5.8)
Significant valvular abnormalities, n (%)	189 (28.7)
Rheumatic valvular changes, n (%)	37 (5.6)

* Plus-minus values are means ± SD. IQR, interquartile range; TRV, tricuspid regurgitant velocity; RAP, right atrial pressure; PASP, pulmonary artery systolic pressure; TAPSE, tricuspid annular plane systolic excursion; LVEF, left ventricular ejection fraction.

### Aetiologies of PH

**Figure 2:**
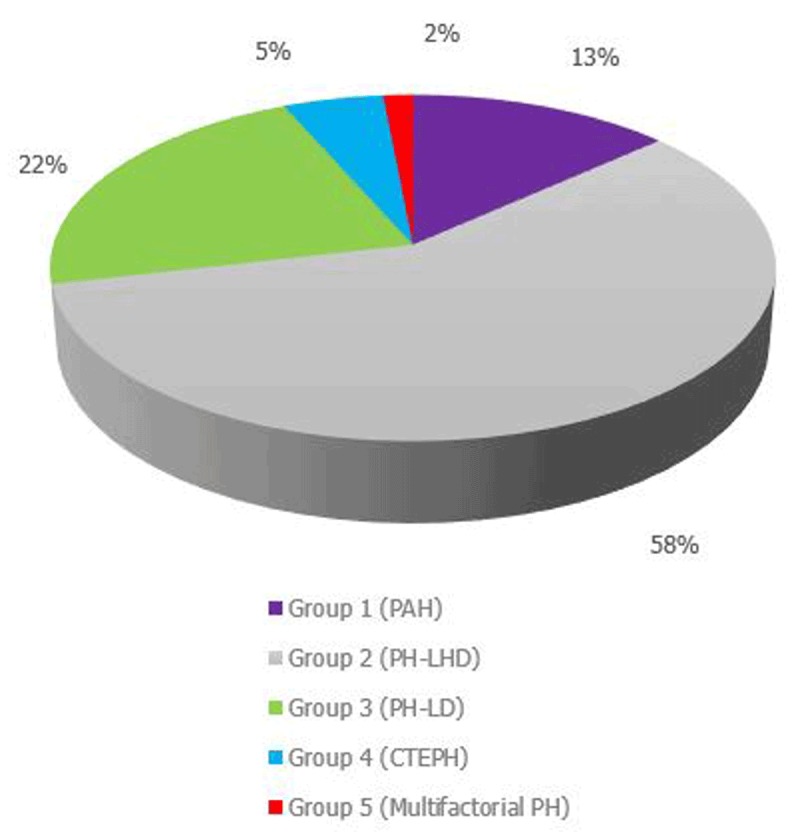
WHO groups of pulmonary hypertension assigned by primary physician. PAH: pulmonary arterial hypertension; PH-LHD: PH due to left heart disease; PH-LD: PH due to lung disease; CTEPH: chronic thromboembolic PH.

Figure 2 below shows the classification of PH as assigned by the patient’s primary physician (n = 546). There was no cause assigned to 113 patients (17%). From the chart, it can be seen that the most common aetiology of PH in this cohort was PH due to left heart disease (58%), followed by PH due to lung disease (22%), pulmonary arterial hypertension (13%) and chronic thromboembolic pulmonary hypertension (5%).

In terms of sub-groups, congenital heart disease (n = 31, 5.7%) was the most common cause of PAH, followed by HIV-associated PAH (n = 18, 3.3%). Heart failure with reduced ejection fraction was the most common cause of PH due to left heart disease (n = 164, 30%). Rheumatic heart disease was a cause of PH in 6.6% of patients. Among those with PH caused by lung disease (PH-LD), chronic obstructive pulmonary disease was the most frequent cause (n = 63, 11.5%), followed by sleep-disordered breathing (n = 23, 4.2%). Table 3 provides a detailed description of the sub-group classification.

**Table 3:** Sub-group classification of pulmonary hypertension.

	Total (N = 546)	%

Group 1	**72**	
Congenital heart disease	31	5.7%
HIV associated PAH	18	3.3%
Connective tissue disease	15	2.7%
Idiopathic PAH	6	1.1%
Portal hypertension	2	0.4%
Group 2	**318**	
HFrEF	164	30.0%
HFpEF	85	15.6%
Rheumatic valvular heart disease	36	6.6%
Other valvular heart diseases	31	5.6%
Others	2	0.4%
Group 3	**122**	
COPD	63	11.5%
Sleep disordered breathing	23	4.2%
Interstitial lung disease	13	2.4%
Post TB bronchiectasis	7	1.3%
Others	14	2.89%
Group 4	**28**	5.1%
Group 5	**8**	
Sickle cell disease	4	0.7%
Others	4	0.7%

PAH, pulmonary arterial hypertension; HFrEF, heart failure with reduced ejection fraction; COPD, chronic obstructive pulmonary disease; HFpEF, heart failure with preserved ejection fraction; TB, tuberculosis. Of the congenital heart disease 16 had ASD, 1 PDA, 1 VSD, 4 Tetralogy of Fallot and 9 others.

### Follow-up and Outcomes

**Figure 3:**
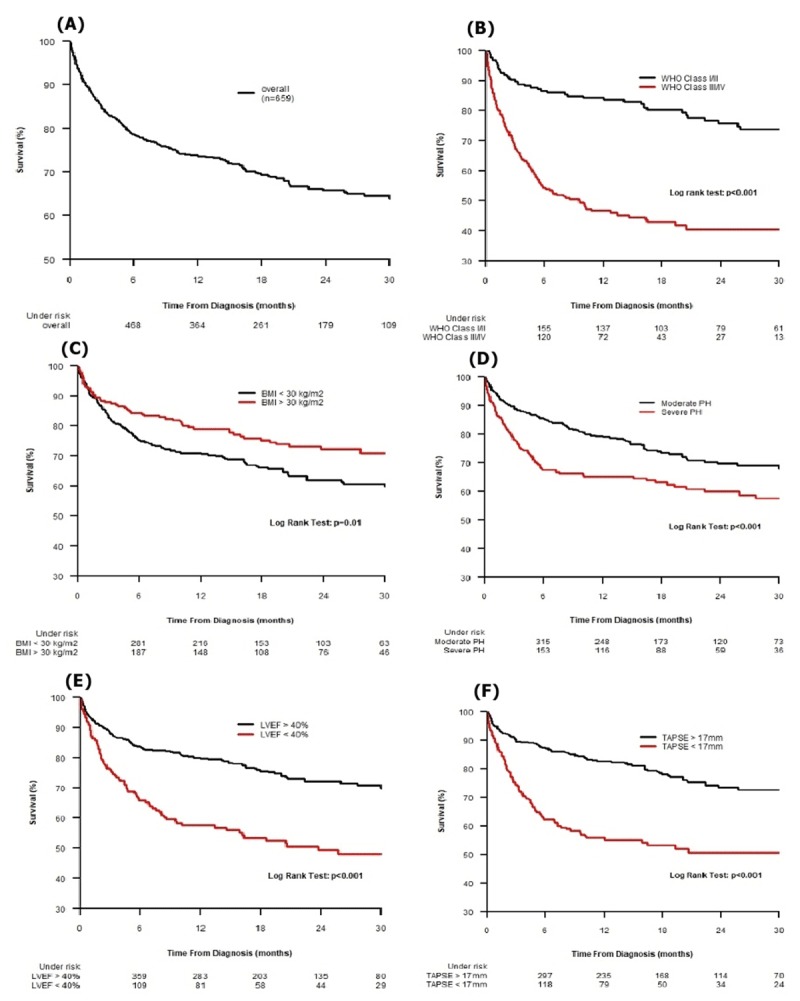
Kaplan-Meier survival estimates for **(A)** Overall cohort; **(B)** by WHO functional class; **(C)** by BMI; **(D)** by severity of PASP; **(E)** by LVEF and **(F)** by TAPSE. WHO, World Health Organization; BMI, Body Mass Index; PH, pulmonary hypertension; PASP, pulmonary artery systolic pressures; LVEF, left ventricular ejection fraction; LV, left ventricular; RV, right ventricular; TAPSE, tricuspid annular plane systolic excursion.

By the end of the study period (median follow-up time: 626 days), 198 patients (30%) had died. As shown in Table 4, the overall survival at 6 months, 1 and 2 years of follow-up was 78.9%, 73.8% and 65.9% respectively. Kaplan-Meier survival curves for the entire cohort by WHO functional class, obesity, PASP, left ventricular dysfunction and right ventricular dysfunction are shown in Figure 3.

**Table 4:** Two-year survival rates.

	Probability of survival (%)

6 months	78.9 ± 1.6
1 year	73.8 ± 1.8
2 years	65.9 ± 2.1

* Plus-minus values are overall survival rate ± SE.

There were statistically significant differences in survival between the 2 groups. The deceased group reported significantly higher PASP, WHO functional class, left ventricular systolic and right ventricular dysfunction (log-rank test p = < 0.001). The estimated survival rates of the PH patients with WHO FC III/IV at 6 months, 1 and 2 years were 54%, 47% and 41% respectively. Patients who were obese (BMI ≥ 30kg/m^2^) at the time of diagnosis had a better overall survival (log rank test P = 0.01).

### Prognostic Factors Associated with Mortality

The relationship between mortality and prognostic factors was assessed in a univariate cox regression model. All factors were prognostic except gender, systemic arterial hypertension and rheumatic heart disease. Upon testing in a multivariate cox regression model, significant valvular heart disease lost its prognostic value. However, age [adjusted HR 1.02, 95% CI (1.01–1.03)], diabetes mellitus [adjusted HR 1.49, 95% CI (1.13–1.98)], WHO functional class III/IV [adjusted HR 3.50, 95% CI (2.47–4.97)], atrial fibrillation [adjusted HR 1.53, 95% CI (1.07–2.17)], severe PH [adjusted HR 1.68, 95% CI (1.27–2.22)], right ventricular dysfunction [adjusted HR 2.61, 95% CI (1.93–3.53)] and left ventricular dysfunction [adjusted HR 2.18, 95% CI (1.64–2.91)] were still independently associated with mortality. Obesity was paradoxically associated with improved survival [adjusted HR 0.68, 95% CI (0.50–0.93)]. The results of the cox regression are shown in Table 5.

**Table 5:** Cox regression analysis.

	Univariate Analysis^¶^		Multivariate Analysis^¥^	

	Unadjusted HR ± 95% CI	p-value	Adjusted HR ± 95% CI	p-value

Age	1.03 (1.02–1.04)	<0.001	1.02 (1.01–1.03)	<0.001
Male Gender	1.23 (0.93–1.63)	0.14	1.17 (0.88–1.54)	0.29
Systemic Arterial Hypertension	1.12 (0.82–1.54)	0.50	0.73 (0.52–1.02)	0.07
Diabetes Mellitus	1.61 (1.22–2.14)	0.001	1.49 (1.13–1.98)	0.005
Obesity (BMI ≥ 30)	0.68 (0.50–0.93)	0.01	0.66 (0.48–0.90)	0.01
WHO Class III/IV	3.53 (2.49–5.00)	<0.001	3.50 (2.47–4.97)	<0.001
Atrial Fibrillation	1.72 (1.22–2.42)	0.001	1.53 (1.07–2.17)	0.02
Severe PH (PASP ≥ 60)	1.68 (1.27–2.22)	<0.001	1.68 (1.27–2.22)	<0.001
RV dysfunction (TAPSE < 17 mm)	2.58 (1.92–3.48)	<0.001	2.61 (1.93–3.53)	<0.001
LV dysfunction (LVEF ≤ 40%)	2.15 (1.62–2.85)	<0.001	2.18 (1.64–2.91)	<0.001
Significant valvular heart disease	1.44 (1.07–1.93)	0.02	1.28 (0.93–1.76)	0.13
Rheumatic heart disease	0.54 (0.24–1.21)	0.13	1.19 (0.50–2.80)	0.70

^¶^ Cox regression performed after confirmation of proportional hazard assumptions using log minus log graphs. ^¥^ A multivariate cox regression model adjusted for confounders (age, gender, race and presence of diabetes mellitus). * P < 0.05 was considered significant. BMI, body mass index; WHO, world health organization; PH, pulmonary hypertension; RV, right ventricle; LV, left ventricle; TAPSE, tricuspid annular plane systolic excursion; LVEF, left ventricular ejection fraction. Interactions for Obesity and Age, Obesity and DM, and Obesity and Gender were performed and no interactions were present.

## Discussion

Knowledge about PH in sub-Saharan Africa is largely drawn from the PAPUCO study [5]. The study was prospective and included participants of different ethnic groups from four African countries (i.e. Cameroon, Nigeria, Mozambique and South Africa) [5]. In this study, a diagnosis of PH was made on the basis of echocardiography—right ventricular systolic pressure >35 mmHg, the absence of pulmonary stenosis and acute right ventricular failure, although the exact definitions of these exclusion criteria variables used in the study were not mentioned in the study protocol [17]. The registry data published in June 2016 examined the clinical profiles, aetiologies and six-month outcomes of patients with PH [5]. However, the prognostic factors of mortality and long-term survival have not been comprehensively examined. Therefore, the present study sought to determine the long-term outcomes and associated factors in a cohort with moderate to severe PH. This retrospective cohort study represents one of the largest studies on pulmonary hypertension (PH) in sub-Saharan Africa (SSA).

The study cohort comprised more females than males, similar to what has been reported in other studies [5]. This may be attributable to the fact that certain aetiologies of PH, such as PAH and rheumatic heart disease, have a strong bias towards females [18,19]. Compared to the PAPUCO study, median age in this group was 18 years higher. This can be explained by the high prevalence of left heart disease in this study, which is highly prevalent among the elderly [1,5]. The majority of the patients were diagnosed late, as illustrated by the high proportion of those in WHO functional class III and IV. This finding is consistent with other African studies on PH [5,20]. In the PAPUCO study, Thienemann et al. reported that two-thirds of patients with PH presented in WHO class III/IV [5]. This may be due to low access to care, healthcare worker education gaps or lack of diagnostic capacity, given low resources [5].

Similar to other studies, PH due to left heart disease (PH-LHD) was the most common, accounting for 30% in this cohort, though at a lower prevalence than the 69% reported in previous studies [5]. This is because our study had a higher prevalence of PH due to lung disease and CTEPH. PH-LHD was majorly attributed to heart failure characterised by reduced ejection fraction, consistent with studies demonstrating high occurrence of systolic heart failure in Africa [21]. It was disconcerting to note that rheumatic valvular disease was also associated with PH in this study, pointing to its endemicity and contribution to the cardiovascular disease burden in this setting. Contrary to other studies, tuberculosis-associated obstructive pulmonary disease was not a significant cause of PH-LD [5,20]. This may be due to mis-classification, since not all patients were evaluated systematically to exclude tuberculosis.

In terms of survival, the findings are similar to those of the PAPUCO study that reported a high rate of mortality at 6 months follow-up [5]. Nonetheless, the overall 3-year survival in this group of patients is 5 to 10% lower than those reported in studies outside Africa [6,22,23]. This may be attributed to the exclusion of patients with mild PH in this study, as well as the barriers to early diagnosis and treatment of PH in this setting [5].

Age was one of the prognostic factors for PH mortality. Older age has been shown to place PH patients at an increased risk of mortality [6,22]. The Giessen Pulmonary Hypertension registry demonstrated that age <50 years predicted survival in all PH groups [22]. A separate study showed that individuals aged ≥65 yrs have high mortality rates [24]. Though systemic arterial hypertension was highly prevalent in our cohort, it had no significant influence on mortality. This is consistent with findings from other heart failure studies in Africa [21].

Several clinical variables were associated with mortality similar to other studies. These included poor functional class (WHO functional class III/IV), diabetes mellitus and atrial fibrillation [6,22,24,25].

The observed reduced mortality in obese patients may support the ‘obesity paradox’ hypothesis. This finding is consistent with that of Zafrir et al., who found that obesity was significantly associated with lower mortality in PH patients (HR 0.2, 95% CI 0.1–0.6; P = 0.004) [26]. In another study, Caceres et al. observed that a higher BMI was associated with a lower relative risk for one year mortality in patients with PH [27]. The ‘obesity paradox’ in this cohort may indeed be due to the effect of higher muscle mass in this cohort of patients rather than the BMI, a finding that was not explored, but has been found in other trials [28]. Recent studies have, however, disputed this. A large population-based study revealed that obesity is associated with shorter longevity and significantly higher cardiovascular risk compared to normal weight [29]. A possible explanation for this conflicting data may be the presence of significant confounding from prevalent disease-related weight loss in studies showing the ‘obesity paradox’ phenomenon [30].

Echocardiographic parameters identified to be independently associated with mortality in PH were PASP, TAPSE and left ventricular ejection fraction. Various studies have demonstrated the link between PH severity and mortality [6,25]. In the Armadale cohort, a higher PASP (>60 mmHg) conferred a 3.29 higher odds of mortality compared to patients with lower PASP [6]. Corciova et al. revealed a strong association between PASP and survival duration (r 0.52, p < 0.001) [25]. Right ventricular dysfunction has been related with poor outcomes in PH [25,31]. Using lower cut-off of less than 17 mm, unlike these authors who used a TAPSE cut-off of 18 mm, we also observed a higher risk of mortality. Similar to Corciova et al., left ventricular systolic dysfunction (LVEF ≤ 40%) was a prognostic factor for mortality [25]. This is attributed to the fact that more than half of patients had PH-LHD [25].

## Limitations

Several limitations of this study need to be acknowledged. The diagnosis of PH was based on echo-derived PASP and not right heart catheterization as recommended. The other important limitation lies in the fact that the aetiologies were entirely based on what was assigned by the primary physician, abstracted from the patients’ medical records. The heterogeneity of our patients was another limitation of the study. Our sample size limited our capacity to do a detailed analysis of each aetiology. Future studies should target specific aetiologies of PH in the region. Lastly, the generalized application of these findings is subject to certain limitations. This was a single-centre study in an urban setting. In the future, it will be important to do a multicentre study that will include health facilities in rural areas as well.

## Conclusion

The study findings clearly indicate that PH is associated with high mortality in African patients. Only about 66% of the patients were alive after two years from the time of diagnosis. Increased mortality risk was associated with diabetes mellitus, atrial fibrillation, WHO FC III/IV, severe PH (PASP ≥ 60 mmHg), right ventricular dysfunction (TAPSE < 17 mm) and left ventricular systolic dysfunction (LVEF ≤ 40%). Obesity was associated with improved survival. On the other hand, the presence of rheumatic heart disease or significant valvular abnormalities had no effect on mortality.
